# 1196. Environmental Contamination of Rooms of Patients Harboring Multidrug-Resistant Organisms

**DOI:** 10.1093/ofid/ofac492.1030

**Published:** 2022-12-15

**Authors:** Alex M Page, Ahmed Babiker, Amanda F Strudwick, Eileen Burd, Sarah W Satola, Michael H Woodworth

**Affiliations:** Emory University School of Medicine, Atlanta, Georgia; Emory University School of Medicine, Atlanta, Georgia; Emory University School of Medicine, Atlanta, Georgia; Emory University School of Medicine, Atlanta, Georgia; Emory University School of Medicine, Atlanta, Georgia; Emory University, Atlanta, GA

## Abstract

**Background:**

Healthcare environmental contamination by patients harboring multidrug-resistant organism (MDROs) is an important source of hospital MDRO transmission. We aimed to determine the MDRO contamination and bioburden of surfaces within hospital rooms of patients with positive MDRO clinical cultures.

**Methods:**

Patients with positive clinical cultures of MDROs (carbapenem resistant *Acinetobacter baumannii* [CRAB], carbapenem resistant *Pseudomonas aeruginosa* [CRPA], extended spectrum cephalosporin and carbapenem resistant Enterobacterales [ESCRE, CRE] and vancomycin resistant *Enterococcus* [VRE]) were identified through daily screening of clinical microbiology results. Patient peri-rectal, inguinal, and wound sampling was performed. E-swab and environmental sampling of room surface composites was performed using environmental sponge wipes. Composite 1 included the TV remote, telephone, call button and bed rails. Composite 2 included the room door handle, IV pole and overbed table. Composite 3 included toileting surfaces. Each composite surface area was no more than 350mm^3^ each. Sponge wipes were expressed in phosphate-buffered saline containing 0.1% Tween 20 using a stomacher. The eluate was concentrated removed leaving ∼ 5mL, and remaining resuspended by vortex. Undiluted suspension was plated on selective MDRO medias and broth. Microbial burden was calculated by summing composites bioburdens. Samples that were only broth positive were given a value of 1 CFU.

**Table 1.**
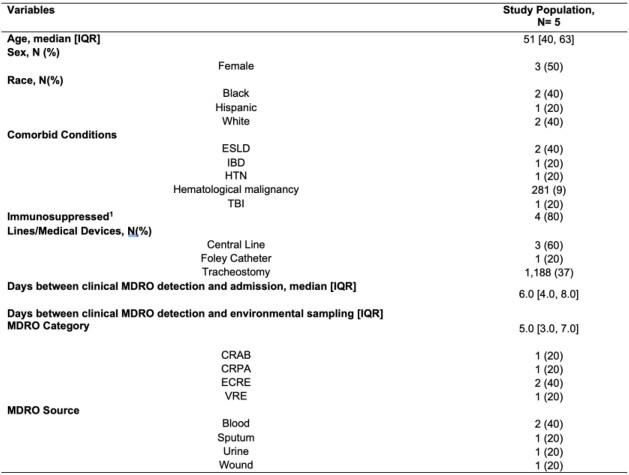
Demographic and Clinical Characteristics of Multidrug-resistant Organisms Positive Patients (n=5)

**Table 2.**
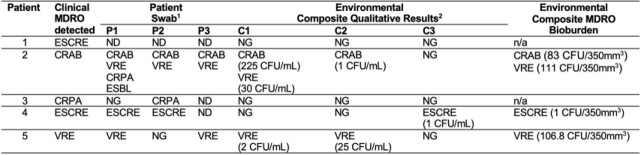
Multidrug-resistant Organism Environmental Contamination and Bioburden of Patients and Environmental Composites

**Results:**

Five patients were included with target MDROs (1 CRAB, 1 CRPA, 2 ESCRE, 1 VRE). Demographic, clinical, and microbiological characteristics are summarized in **Table 1**. The same MDRO was detected in the environment of 60% (3/5) patients. Additional MDROs other than the clinical culture were cultured from patient and composite sites swabs (**Table 2)**. Antibiotic susceptibility data confirmed similarity between patient and environmental isolates and identified additional MDROs present (**Table 3**,**4**). Additional patient enrollment and WGS isolates from clinical culture, patient and environmental isolates is ongoing.

**Table 3.**
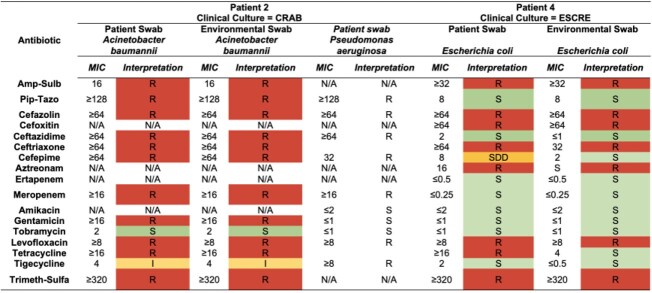
Antimicrobial Susceptibility Profile of Isolates from Patients with Concordant Clinical and Environmental MDROs

**Table 4.**
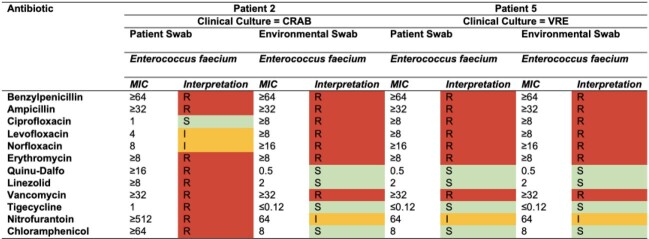
Antimicrobial Susceptibility Profile of Concordant Clinical and Environmental MDROs

**Conclusion:**

Concordance of clinical isolates with environmental isolates suggest that decolonization interventions could reduce environmental bioburden

**Disclosures:**

**All Authors**: No reported disclosures.

**Table 1.** Demographic and Clinical Characteristics of Multidrug-resistant Organisms Positive Patients (n=5)

Abbreviation: CRAB: carbapenem-resistant Acinetobacter baumanii, CRPA: carbapenem-resistant Pseudomonas aeruginosa, ESCRE: extended spectrum cephalosporin-resistant Enterobacterales, ESLD: end-stage liver disease, IBD: inflammatory bowel diseases, HTN: hypertension, MDRO: multidrug resistant organism, TBI: traumatic brain injury, VRE: vancomycin resistant enterococcus

1- Immunosuppressed defined as solid organ transplant, bone marrow transplant within 12 months, chemotherapy within 6 months, HIV and CD4 <200, any immunomodulatory agent within the past 30 days, at least 10 mg of corticosteroid for >14 days

**Table 2.** Multidrug-resistant Organism Environmental Contamination and Bioburden of Patients and Environmental Composites

Abbreviation: CRAB: carbapenem-resistant Acinetobacter baumannii, CRPA: carbapenem-resistant Pseudomonas aeruginosa, ESCRE: extended spectrum cephalosporin-resistant Enterobacterales, VRE: vancomycin resistant Enterococcus, ND: not done, NG: no growth, N/A; not applicable

1Patient Swab P1: peri-rectal, P2: wound or tracheostomy, P3: inguinal

2Environmental Swab C1: TV remote, telephone, call button and bed rails; Composite 2 room door handle, IV pole and overbed table; Composite 3: toileting surfaces

**Table 3.** Antimicrobial Susceptibility Profile of Isolates from Patients with Concordant Clinical and Environmental MDROs

Abbreviation: CRAB: carbapenem-resistant Acinetobacter baumannii, ESCRE: extended spectrum cephalosporin-resistant Enterobacterales, MIC: minimum inhibitory concentration, N/A; not applicable

**Table 4.** Antimicrobial Susceptibility Profile of Concordant Clinical and Environmental MDROs

Abbreviation: CRAB: carbapenem-resistant Acinetobacter baumannii, MIC: minimum inhibitory concentration, N/A; not applicable

, VRE: vancomycin resistant enterococcus

